# Management of the axilla in T1-2N1 breast cancer

**DOI:** 10.1038/s41523-022-00432-y

**Published:** 2022-05-30

**Authors:** Mahmoud El-Tamer, Tibor Kovacs

**Affiliations:** 1 Breast Service, Department of Surgery, Memorial Sloan Kettering Cancer Center, New York, NY USA; 2 Breast Institute, Jiahui International Hospital, Shanghai, China; 3 Guy’s and St. Thomas’ NHS Foundation Trust, London, UK

**Keywords:** Breast cancer, Chemotherapy

## Abstract

In the setting where the ongoing evolution of management of the axilla in breast cancer is being driven by better understanding of different sub-types of the disease, and of how these respond to chemotherapy, here we discuss management of the axilla in breast cancer patients who present with T1 or T2 N1 disease, while making the distinction between triple negative and HER2 positive tumors as one group, and hormone receptor positive/HER2 negative tumors as a second group.

The management of the axilla in breast cancer has been gradually changing over time. This change is driven by better understanding of breast cancer metastasis and different sub-types of the disease, and their response to chemotherapy. Furthermore, with the increase in survival from breast cancer, patients report a significant impact on their quality of life from the long-term side effects of axillary lymph node dissection.

In this commentary, we discuss the management of the axilla in breast cancer patients who present with T1 or T2 N1 disease. With our better understanding of the different subtypes of breast cancer, we address triple negative and human epidermal growth factor receptor 2 (HER2) positive tumors separately from hormone receptor positive/HER2 negative tumors.

## Management of the axilla in T1-2N1, triple negative, and HER2 amplified breast cancers

It is customary nowadays to deliver chemotherapy as a first line of treatment in all patients with triple negative, and HER2 amplified tumor subtypes who present with positive nodes. Multiple prospective and retrospective studies have shown that a significant number of these patients will achieve a pathologic complete response (pCR) in the axillary nodes. Table 1 lists the pCR rates of these 2 subtypes in 4 different studies^1–4^. Chemotherapy for triple-negative tumors has been reported to achieve a pCR rate of around 50%. In HER2 amplified tumors, the pCR rate is even higher, varying between 49% and 65%. Furthermore, the axillary pCR rate has been shown to be higher among HER2 positive tumors when the hormone receptors are negative, as compared to those who are hormone receptor positive^5^.

**Table 1:** Nodal pathologic complete response in triple-negative/HER2 positive breast cancers, and in hormone receptor-positive and HER2 negative breast cancers.

Study	No. of patients (stage)	HR positive/HER2 negative	TNBC	HER2 positive
Boughey 2013	756 (pN+)	21%	49%	65%
Kim 2015	415 (pN+)	29%	54%	49%
Montagna 2020	573 (pN+)	20%	44%	63.3%
Simons 2019	139 (pN+)	7.4%	44%	74%

*HR* hormone receptor, *N* node, *TNBC* triple-negative phenotype, *HER2* human epidermal growth factor receptor 2.

There is no known value for an axillary lymph node dissection in patients who have achieved pCR. Imaging modalities may reflect nodal pathologic response, but are not highly sensitive and are not predictive enough to preclude surgical staging. The sensitivity of ultrasound, MRI, and PET/CT to identify residual lymph node disease has been reported to be 70%, 61%, and 63%, respectively^6–8^.

The role of a sentinel lymph node biopsy in predicting pathologic response of the axilla has been extensively evaluated in the literature in multiple prospective studies. Sentinel lymph node biopsy after neoadjuvant chemotherapy has a low false-negative rate with dual mapping and the harvesting of 3 or more sentinel nodes. Tee and colleagues conducted a meta-analysis comprising 13 studies, in which all patients had pathologic confirmation of a positive axillary node prior to initiation of chemotherapy. A sentinel lymph node biopsy was performed after chemotherapy, together with a backup axillary lymph node dissection, in all studies. Most of the studies were prospective (12 of 13). The meta-analysis included 1921 patients. The false-negative rate of the sentinel node was 14%. Dual mapping had an 11% false-negative rate, compared with 19% for single mapping. The number of harvested sentinel nodes was inversely proportional with the false-negative rate; in patients with 3 or more sentinel nodes identified, the false-negative rate fell to 4%^9^.

At Memorial Sloan Kettering Cancer Center (New York, NY, USA), sentinel lymph node biopsy was adopted in all patients with positive nodes who were rendered clinically negative after chemotherapy. Dual-agent mapping, and a minimum of 3 nodes harvested, were prerequisites for a complete sentinel node procedure. In 573 patients between 2013 and 2019, 3 or more sentinel nodes were successfully identified with dual mapping in 93%. The sentinel nodes were negative in 41% of the patients, and all were spared an axillary lymph node dissection^3^.

Sentinel lymph node biopsy can reliably predict the pathologic status of the axilla when 3 or more sentinel nodes are identified with the use of dual-mapping techniques. Even more important than the false-negative rate, however, is the local control of the axilla. The Memorial Sloan Kettering Cancer Center study reported on regional control of the axilla with dual-mapping following a negative sentinel lymph node biopsy after neoadjuvant chemotherapy^10^. Among 234 patients with 3 or more negative sentinel nodes without an axillary dissection and a median follow-up of 40 months, 13 patients developed distant metastasis and only 1 patient developed local recurrence. An axillary and local recurrence was identified in only 1 patient who refused radiation therapy. These data are supportive of the reliability of a sentinel node procedure after neoadjuvant chemotherapy in patients who became clinically node negative after neoadjuvant chemotherapy when proper techniques are used (Box 1).

The American College of Surgeons Oncology Group (ACOSOG) Z-1071 trial has demonstrated a decrease in the false-negative rate when the positive node is clipped and retrieved after neoadjuvant chemotherapy, confirming pathologic complete response. We do not recommend clipping the node for the following reasons: (1) the clip is lost in 3% of patients^11^; (2) the clip is localized in only 95% of the patients and is retrieved in 92% of those localized. Hence, the success rate of identifying the clip is limited to 85% of patients^12^. However, with dual mapping, 3 sentinel nodes are identified in 93% of patients^3^; (3) The false-negative rate of the clipped node is 7%^11^, whereas it is 4% when 3 negative sentinel nodes are identified without relying on the clipped node^9^; (4) More importantly, the Memorial Sloan Kettering Cancer Center group reported no axillary recurrence without clipping^10^. Similarly, the European Institute of Oncology group reported a 1.6% axillary recurrence without clipping the positive node, albeit using less-stringent criteria than the Memorial Sloan Kettering Cancer Center group^13^.

The practice of performing a sentinel node procedure prior to neoadjuvant chemotherapy should be abandoned. It is associated with a significant loss of a predictor of response in the axilla. A repeat sentinel lymph node biopsy at the completion of chemotherapy is unreliable, as the detection rate is 60.8% and the false-negative rate is 51.6% as demonstrated in the SENTINA trial^14^. This practice would commit patients with initially positive nodes to a completion axillary node dissection after neoadjuvant chemotherapy.

The standard of care for positive sentinel lymph node biopsy after neoadjuvant chemotherapy is completion axillary lymph node dissection. In patients with a negative sentinel node on frozen section and a positive sentinel node on definitive pathology evaluation, we continue to recommend a completion axillary lymph node dissection. The rate of residual positive disease in the axilla when the frozen section was falsely negative has been reported to be 64%^15^. Currently, the Alliance A011202 trial is evaluating the role of a completion axillary lymph node dissection in patients with positive sentinel nodes after neoadjuvant chemotherapy. The study randomizes patients with clinical T1-3N1 disease who are found to have a positive sentinel lymph node biopsy in 2 arms. The first arm is the standard-of-care arm, which includes a completion axillary lymph node dissection followed by radiation therapy to the regional nodes, the breast, or the chest wall, depending on the surgical procedure (mastectomy or breast conservation). In the second arm, the axillary lymph node dissection is omitted and the patient receives only radiation therapy to the regional nodes, the breast, or the chest wall.

**Fig. 1:**
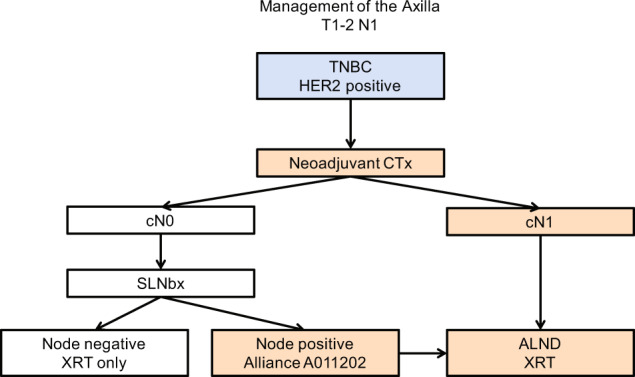
Suggested management of the axilla with T1/T2 N1 disease who are triple negative or HER2 amplified. (Patients who are cN0 after neoadjuvant chemotherapy and have a positive sentinel node may be candidates for the Alliance A011202 trial, which randomized patients to a full axillary lymph node dissection versus none; all patients will receive radiation therapy). TNBC triple-negative breast cancer, HER2 human epidermal growth factor receptor 2, CTx chemotherapy, SLNB sentinel lymph node biopsy, XRT radiation therapy, ALND axillary lymph node dissection.

To summarize management of the axilla in T1-2N1, triple negative, and HER2 amplified breast cancers: (1) we recommend abandoning the practice of performing a sentinel lymph node biopsy prior to neoadjuvant chemotherapy; (2) the reliability of sentinel node biopsy after neoadjuvant chemotherapy requires the use of dual-agent mapping technique and identifying a minimum of 3 or more sentinel nodes (the false-negative rate can be as low as 4%); (3) we do not recommend clipping or tagging the positive node prior to neoadjuvant chemotherapy, as we find that sentinel lymph node biopsy without node clipping is a reliable technique in predicting the status of the axilla after neoadjuvant chemotherapy; (4) the success rate of identifying 3 or more sentinel nodes after neoadjuvant chemotherapy is 93%; and (5) completion axillary lymph node dissection is the standard of care for all patients in whom the sentinel node is positive or in whom there is a failure to identify 3 or more sentinel nodes (consider entering patients with residual positive sentinel nodes into the Alliance A011202 trial) (Fig. 1).

Box 1 Criteria for SLNB after NAC in patients with node-positive disease at presentation Criteria for sentinel lymph node biopsy after neoadjuvant chemotherapy in patients with node-positive disease at presentation:

- Limited to patients with N1 disease.
- Rendered clinically node negative after NAC.
- A well-versed team in sentinel node procedures.
- Use of dual-mapping techniques.
- Resect all sentinel nodes and harvest 3 or more nodes.
- Pathologic confirmation of complete response in the lymph node.
- Look for treatment effect in the sentinel nodes.

## Management of the axilla in T1-2N1, hormone receptor-positive/HER2 negative breast cancers

The rate of pCR in axillary nodes for hormone receptor-positive/HER2 negative tumors varies between 7.4% and 29.0%. Table 1 compares the nodal pCR in different tumor subtypes^1–4^.

Three important studies have shaped the creation of guidelines for the management of the axilla in hormone receptor-positive/HER2 negative tumors after chemotherapy: The Treatment for Positive Node, Endocrine Responsive Breast Cancer (RxPONDER) trial, the ACOSOG Z0011 trial, and the After Mapping of the Axilla: Radiotherapy or Surgery (AMAROS) trial.

The RxPONDER study accrued patients with estrogen receptor and/or progesterone receptor positive (>1%), HER2 negative breast cancers with 1-3 positive lymph nodes, and an Oncotype DX (Exact Sciences, Redwood City, CA) score of 0–25. The patients were randomized to receive chemotherapy and endocrine therapy, or endocrine therapy alone. The study showed that adjuvant chemotherapy has no impact on invasive disease-free survival in postmenopausal women. However, the study did show a clear benefit for premenopausal patients^16^.

The ACOSOG Z0011 trial randomized breast cancer patients who were clinically node negative with T1-2 tumors and found to have 1-2 positive sentinel nodes to an axillary lymph node dissection or no further axillary procedure. It concluded that a full axillary lymph node dissection could be safely omitted in that group of patients, as there was no difference in locoregional recurrence, disease-free survival, or overall survival^17^.

The AMAROS trial accrued patients with stage cT1-2N0M0 primary breast cancer. Those with positive sentinel nodes were randomized to an axillary lymph node dissection or axillary radiation therapy. At a median follow-up of 10 years, both of these arms showed no difference in regional recurrence, distant metastasis, or survival^18,19^.

For hormone receptor-positive/HER2 negative T1-2 tumors, the decision on chemotherapy is dependent on the menopausal status of the patient as well as on the clinical examination of the axilla.

It is clear that patients with N1 disease who are premenopausal require chemotherapy irrespective of their Oncotype DX score. In postmenopausal patients, the RxPONDER trial showed no benefit from chemotherapy in postmenopausal patients with 1-3 positive nodes. The ACOSOG Z0011 and AMAROS trials apply to patients who are clinically node negative; ACOSOG Z0011 included only patients who underwent breast-conservation therapy, while AMAROS included patients with breast conservation or mastectomy.

Based on the above, the management of hormone receptor-positive/HER2 negative T1/2 N1 patients depends on their menopausal status, clinical exam of the axilla, surgical procedure, Oncotype DX score, and number of positive nodes.

**Fig. 2:**
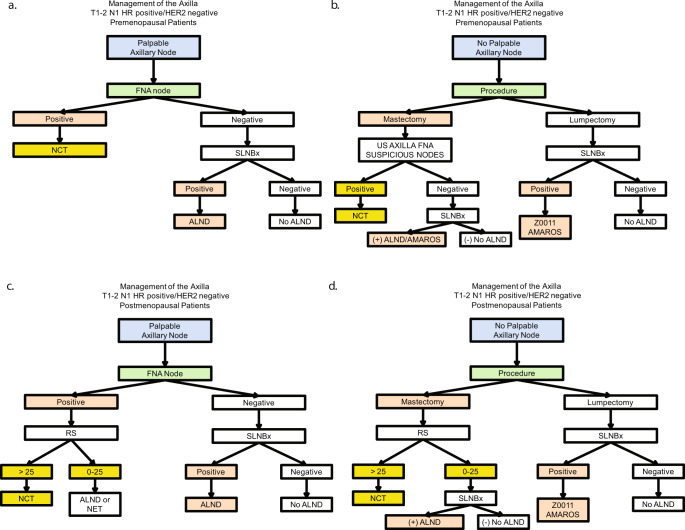
Suggested management of the axilla with T1/T2 N1 disease who are hormone receptor positive/HER2 negative. **a** Management of the axilla for premenopausal patients with hormone receptor positive/HER2 negative T1-2 N1 patients with palpable axillary nodes. **b** Management of the axilla for premenopausal patients with hormone receptor positive/HER2 negative T1-2 N1 patients with no palpable axillary nodes. **c** Management of the axilla for postmenopausal patients with hormone receptor positive/HER2 negative T1-2 N1 patients with palpable axillary nodes. **d** Management of the axilla for postmenopausal patients with hormone receptor positive/HER2 negative T1-2 N1 patients with no palpable axillary nodes. TNBC triple negative breast cancer, HER2 human epidermal growth factor receptor 2, CTx chemotherapy, SLNB sentinel lymph node biopsy, XRT radiation therapy, ALND axillary lymph node dissection, HR hormone receptor, FNA fine-needle aspiration, NCT neoadjuvant chemotherapy, RS Oncotype DX recurrence score.

Premenopausal patients with clinically palpable axillary nodes should have a cytologic or pathologic evaluation of the palpable node to confirm metastatic disease. Patients with positive palpable nodes are recommended neoadjuvant chemotherapy. Those with negative nodes should have a sentinel node procedure that will dictate further management of the axilla as seen in Fig. 2a.

In clinically node-negative premenopausal patients, the surgical procedure (mastectomy or lumpectomy) determines the management pathway. When a lumpectomy is planned, patients with positive sentinel nodes may be managed based on the ACOSOG Z0011 or AMAROS trials. The patients who are undergoing a mastectomy have not been included in the ACOSOG Z0011 trial, and the benefit of chest wall radiation may not be justified in all patients. We recommend a sonographic evaluation of the axilla with fine-needle aspiration (FNA) of suspicious nodes. Patients with proven nodal metastasis are started on neoadjuvant chemotherapy, while those with negative FNA are candidates for upfront surgical intervention. Fig. 2b summarizes the suggested outline of treatment.

In postmenopausal patients with clinically palpable lymph nodes, the result of an FNA of the axillary node will determine the clinical pathway. Patients with negative cytology will undergo a sentinel lymph node biopsy with complete axillary lymph node dissection if the sentinel node is positive, as the number of positive nodes will dictate if they can be managed as per the RX-PONDER trial. In patients with positive FNA, those with Oncotype DX recurrence scores >25 will be candidates for neoadjuvant chemotherapy. Those with Oncotype DX recurrence scores ≤25 may be candidates for a full axillary lymph node dissection on neoadjuvant endocrine therapy as per Fig. 2c.

In postmenopausal patients who do not have palpable axillary nodes, the type of surgical procedure will determine the clinical pathway. Those undergoing a lumpectomy are clearly candidates for the ACOSOG Z0011 or AMAROS trials if they fit their criteria; hence, they should undergo a sentinel node procedure and be treated accordingly. For those undergoing a mastectomy, the recurrence score is helpful in determining the need for neoadjuvant chemotherapy; a need that would be limited for those with an Oncotype DX score >25. Those with Oncotype DX scores ≤25 will undergo a sentinel lymph node biopsy and completion node dissection only if the sentinel node is positive, as shown in Fig. 2d.

To summarize the management of the axilla in hormone receptor-positive/HER2 negative tumors: (1) sentinel lymph node biopsy is standard for cN0 patients; (2) sentinel lymph node biopsy is standard for patients with cT1-2 tumors and 1-2 positive nodes who are undergoing a lumpectomy and whole breast irradiation; and (3) axillary dissection is recommended when a patient has palpable positive nodes if no neoadjuvant chemotherapy is planned, when a patient has >2 positive sentinel nodes, and when residual positive nodes are present after neoadjuvant chemotherapy.

### Reporting summary

Further information on research design is available in the Nature Research Reporting Summary linked to this article.

## Supplementary information

Reporting Summary Checklist

## Data Availability

Available by contacting the corresponding author upon request.
